# Systematic review of the surgical management of rotator cuff repair with an augmentative patch: a feasibility study protocol

**DOI:** 10.1186/s13643-018-0851-1

**Published:** 2018-11-13

**Authors:** Gemma Greenall, Andrew Carr, David Beard, Jonathan Rees, Amar Rangan, Naomi Merritt, Melina Dritsaki, Navraj S. Nagra, Mathew Baldwin, Sally Hopewell, Jonathan A. Cook

**Affiliations:** 1 Nuffield Department of Orthopaedics, Rheumatology and Musculoskeletal Sciences, University of Oxford, Oxford, UK; 2 The James Cook University Hospital, South Tees Hospitals NHS Foundation Trust, Middlesbrough, UK

**Keywords:** Patch, Systematic review, Rotator cuff tear, Tendon injury, Shoulder surgery, Tissue scaffold, Surgical mesh, Acellular dermal matrix

## Abstract

**Background:**

Shoulder pain is a common problem in the general population and is responsible for prolonged periods of disability, loss of productivity, absence from work and inability to carry out household activities. Rotator cuff problems account for up to 70% of shoulder pain problems and are the third most prevalent musculoskeletal disorder after those occurring in the lower back and neck. Rotator cuff surgery has high failure rates (25–50% within 12 months), and as a result, there is a pressing need to improve the outcome of rotator cuff surgery. Patch augmented surgery for rotator cuff repairs has recently been developed and is increasingly being used within the UK National Health Service. Patch augmented surgery could lead to a dramatic improvement in patient and surgical outcomes, but its clinical and cost effectiveness needs rigorous evaluation. The existing evidence on the use of patches may be at risk of bias as currently only a small number of single-centre comparative studies appear to have been carried out. Additionally, it is unclear for which patches a clinical study (comparative and non-comparative) has been conducted. This paper outlines the protocol for a systematic review intended to summarise the best available clinical evidence and will indicate what further research is required.

**Methods:**

Electronic databases (Medline, Embase and Cochrane) will be systematically searched between April 2006 and the present day for relevant publications using a specified search strategy, which can be adapted for the use in multiple electronic databases, and inclusion criteria. Screening of both titles and abstracts will be done by two independent reviewers with any discrepancies resolved by a third independent reviewer. Data extraction will include information regarding the type of participants, type of intervention and outcomes including but not limited to shoulder-specific function and pain scores, patch-related adverse events and type of study. The results will be summarised in a narrative review where qualitative analysis is not possible.

**Discussion:**

This review aims to collate the current evidence base regarding the use of patches to augment rotator cuff repair. The results of this review will help to develop, using consensus methods, the design of a definitive randomised trial assessing the clinical and cost-effectiveness of a patch to augment surgical repair of the rotator cuff that is both acceptable to stakeholders and is feasible.

**Systematic review registration:**

CRD42017057908

**Electronic supplementary material:**

The online version of this article (10.1186/s13643-018-0851-1) contains supplementary material, which is available to authorized users.

## Background

Shoulder pain is a common problem in the general population and is responsible for prolonged periods of disability, loss of productivity, absence from work and inability to carry out household activities. Rotator cuff problems relate to the tendons and muscles surrounding the shoulder joint. They account for up to 70% of shoulder pain problems and are the third most prevalent musculoskeletal disorder after those occurring in the lower back and neck [1].

A challenging but common rotator cuff problem is a rotator cuff tendon tear, found in about 25% of people aged 70 and above [1]. Symptoms include pain, weakness, lack of shoulder mobility and sleep disturbance. Initial management is conservative and includes rest with simple pain management using paracetamol and non-steroidal anti-inflammatory drugs. Approximately 40% of patients will continue to experience pain despite conservative management, and many will require surgery to repair the tear [2, 3].

Rotator cuff surgery has high failure rates (25–50% within 12 months) [4, 5] and is expensive, invasive and inconvenient to patients. Re-operation is also sometimes necessary. Around 9,000 rotator cuff repairs are performed each year in the National Health Service (NHS) in England, at a cost of £6,628 per operation (£60 million per year), and this number is still growing [6, 7]. There is a pressing need to improve the outcome of rotator cuff surgery [8].

Various surgical approaches have been tried unsuccessfully to improve the outcome of rotator cuff repair [6, 9]. Patch augmented surgery for rotator cuff repair has recently been developed and anecdotally would appear to be increasingly used within the UK NHS. Using a patch to provide a support structure or ‘scaffold’ for the repair, to improve the fixing of the tendon to the bone and tendon healing, has provided promising results [10–12]. The patch is surgically sutured on top of the tendon-to-bone repair to strengthen the repair and aid the healing process, thereby reducing the likelihood of failure and improving patient outcomes [13].

Patches have been made using different materials (human/animal heart, skin or intestine tissue, and completely synthetic materials) and processes (e.g. woven or mesh approaches) and to different sizes. Some have been designed specifically for rotator cuff repair surgery or can be tailored in size and shape for use in this operation, whereas others were developed for other soft-tissue contexts (e.g. anterior cruciate ligament reconstruction in the knee or for hernia repair). They can also be designed to be absorbable, avoiding the possibility of later surgical complications or surgical removal [14]. A number of pre-clinical (in vivo and in vitro mechanical properties) studies have been conducted, evaluating various patches, which have had positive findings [15].

Patches differ in how they respond to tendon tissue and their mechanical properties [16]. Recent advances include the development of electrospun materials [12] and exploration of the concurrent use of growth factors. Electrospun materials have a structure that closely resembles the surrounding tissue, and they provide biological cues to encourage cell growth and tissue healing [12]. The aim of these and other biomimetic materials is to avoid adverse immunological responses, which some tissue-based patches have provoked [17]. Augmenting surgical repair with a patch may also enable the repair of tears that are currently considered unrepairable [10, 14, 18–20].

There is a pressing need to improve the surgical options available for rotator cuff repairs in order to improve tendon healing and patient outcomes [8]. Various other approaches have been attempted to improve the outcome of this surgery without success. Innovative surgical intervention requires evaluation for efficacy and safety. Whilst some studies have been conducted on the use of a patch for rotator cuff repair, there would appear to have only been a small number of single-centre comparative studies (predominantly based in North America) carried out [10, 17, 21, 22]; additionally, they seem to have evaluated only a subset of the available patches.

Patch augmented surgery could lead to a dramatic improvement in patient and surgical outcomes, but the clinical and cost-effectiveness of the intervention needs rigorous evaluation. This protocol outlines the objectives of the systematic review and the methods used to identify the relevant current evidence base. It is in line with the reporting standards set out in the PRISMA-P checklist [see Additional file 1: PRISMA-P checklist].

## Objectives

The aim of this systematic review is to identify and critically appraise studies reporting on the clinical use of patch augmented surgical repair of a rotator cuff tear in terms of clinical effectiveness and safety. There is scope within this systematic literature review to also identify the most clinically effective and safe candidate patches for use in a future definitive randomised controlled trial (RCT).

## Methods/design

### Criteria for considering studies for this systematic review

In order to search for and identify the relevant studies, a scoping search was initially undertaken to inform the definitions used in the final search strategy and corresponding inclusion criteria. The finalised criteria applied to address the research question are as follows:

#### Types of participants

Studies evaluating adults (≥ 18 years) who require surgical repair of a rotator cuff tear will be included. No restrictions will be applied to comorbidities (diabetes, heart disease etc.), the type of rotator cuff tear (partial or full thickness), tendon involvement (supraspinatus, infraspinatus, teres minor or subscapularis) or based on whether the tear is a first occurrence or recurrence. Patients receiving surgical treatment for additional pathology such as a fracture or osteoarthritis, alongside a rotator cuff tear, will not be excluded, providing the principal use of the patch is for the repair of the rotator cuff tear.

Studies where there is a mixed population of patients receiving a patch for rotator cuff repair or another indication such as patella or bicep repairs will be excluded if the results are not reported for each individual indication.

#### Types of intervention/comparators

All studies where at least one treatment arm includes the use of either commercially or non-commercially available patches to augment rotator cuff surgery will be included. Studies may have a single intervention group or may have two or  more interventions groups. There is no restriction with regard to any comparison group (e.g. a study could compare patch A vs patch B, or a patch versus a drug or other kind of intervention). For the purposes of this systematic review, a patch is defined as an implantable human, synthetic, or animal material which is used with the aim of improving tissue healing and/or patient outcome via some form of mechanical support. There will be no exclusions based on the type (synthetic or non-synthetic) or the source of the patch (e.g. human, porcine, bovine or equine); studies of suture and anchors used in isolation will not be eligible for inclusion. There will be no restriction on the type of surgery received or the grade or experience of the surgeon completing the surgery.

Studies reporting on non-relevant interventions only, such as drug therapy or physiotherapy, will be excluded; however, these interventions may, as noted above, be used in a comparator group or used in addition to a patch in the same intervention group within an eligible study.

#### Types of outcome measures

There will be no exclusions made based on the outcomes reported in the identified studies. All outcomes at all time points, regardless of follow-up, will be of interest to this review.

The primary outcomes of interest include, but are not limited to:

(i) Shoulder-specific function and pain scores

Measured using a validated scale such as Oxford Shoulder Score (OSS), American Shoulder and Elbow Surgeon Score (ASES), Disabilities of the Arm Shoulder and Hand (DASH) questionnaire, Constant scale, PENN shoulder score, Shoulder Pain and Disability Index (SPADI), Simple Shoulder Test (SST) or University of California at Los Angeles scale (UCLA).

(ii) Patch-related adverse events (AEs)

Patch-related adverse events whether confirmed as due to the patch or only suspected to be (e.g. severe immune responses).

The secondary outcomes will include but not be restricted to the following:

(iii) Shoulder pain outcomes

Single-item shoulder pain measures to be assessed by validated assessment tools such as the visual analog scale (VAS), Likert pain scale and other validated or non-validated dichotomous, categorical or ordinal assessments or patient-reported outcomes (PROs).

(iv) Health-related quality of life

Including but not limited to overall health-related quality of life (HRQoL) as assessed by EuroQoL-5D (EQ-5D), Short Form-36 (SF36) and Health Utilities Index (HUI).

Other secondary outcomes include:

Recurrence of rotator cuff tear and failure to heal assessed radiographically, revision rates of the surgery, time to surgical revision and patient satisfaction as defined by the included studies.

#### Types of studies

This review will consider all relevant randomised controlled trials (RCTs); however, due to the low use of patch augmented surgeries in practice, there is a paucity of such studies. Therefore, in addition to RCTs, the review will also include non-randomised studies (comparative and single intervention  group) involving ≥ 5 patients. No language restrictions will be applied, and methods of translation will be explored for any non-English included studies that are identified.

In vitro and animal studies will be excluded in addition to review articles, editorials and single case studies. Initial scoping searches did not identify any economic evaluations relating to patch use in shoulder augmentation and therefore will not be included for the purposes of this systematic review. Related economic evaluations will be flagged summarised narratively.

### Search methods for the identification of studies

A search strategy was developed [see Additional file 2: Embase search strategy] in Embase and was adapted for other electronic databases. The searches will be conducted between the dates of April 2006 to the present day, as a previous Cochrane review [9] has identified all relevant key publications prior to April 2006. The following electronic databases will be searched via the OVID and Cochrane Library platforms using the predefined search strategy:

(i) The Cochrane Library, incorporating:
  Cochrane Central Register of Controlled Trials (CENTRAL)
  Cochrane Database of Systematic Reviews
  Database of Abstracts of Reviews of Effects (DARE)
  The Health Technology Assessment Database
  NHS Economic Evaluation Database (NHS EED)
(ii) Ovid MEDLINE(R) In-Process & Other Non-Indexed Citations and Ovid MEDLINE(R), 1946 to present.
(iii) Ovid Embase, 1980 to present

Reference lists of included studies and relevant systematic reviews identified from the Cochrane database of systematic reviews, DARE and the health technology assessment database will be scanned in addition to the reference list of the previously identified Cochrane review [9], and one unpublished and two published systematic reviews assessing patch augmented rotator cuff surgery [11, 23] that were identified during scoping searches.

Other resources will include contacting experts known to have an interest in using patches to augment surgery, contacting authors of key studies already identified during scoping searches, requesting information of relevant research being conducted by the companies working to produce patches and using the surveys developed and distributed as part of the Patch Augmented Rotator Cuff Surgery (PARCS) feasibility study.

Electronic trial registries and regulatory body websites will also be searched to identify any relevant ongoing clinical studies, new systematic reviews or health technology assessments involving patch augmented rotator cuff surgery, these sources will include:

(i) The World Health Organization (WHO) network of primary registries (which includes the ISRCTN.org, EU Clinical Trials Register and Australian New Zealand Clinical Trials Registry)
(ii) Clinicaltrials.gov
(iii) PROSPERO registry
(iv) Centre for Reviews and Dissemination (CRD) database
(v) Medicines and Healthcare products Regulatory Agency (MHRA)
(vi) Food and Drug Administration (FDA) agency

### Selection of studies

Two researchers will screen all titles and abstracts identified from the search strategy independently. Full reports will be obtained if the initial screening indicates that the identified studies are potentially relevant. Full reports that meet the inclusion criteria will be included in the review. Reasons for exclusion will be recorded at each stage and detailed in a PRISMA (Preferred Reporting Items for Systematic Reviews and Meta-Analyses) flow diagram. A third independent reviewer will help to resolve any discrepancies or disagreements if they arise.

### Data extraction and management

A standard data extraction table will be used to extract relevant data under key headings such as study design, patient population, baseline characteristics and surgical characteristics such as the technique and surgeon conducting the intervention, the type of intervention received and all primary and secondary outcomes of the included studies.

Data for relevant outcomes will be extracted where available, and an attempt will be made to contact authors of studies to obtain additional or missing data.

### Assessment of risk of bias in included studies

The risk of bias will be assessed by two independent reviewers (NN, MB). Any discrepancies will be discussed with a third reviewer (GG) and resolved based on unanimous decision. RCTs will be assessed using the tool provided by the Cochrane Collaboration [24]. It assesses studies according to six pre-defined domains: selection bias, performance bias, detection bias, attrition bias, reporting bias and other bias. Each domain will be rated as a ‘low’, ‘high’ or an ‘unclear’ risk of bias before assessing the study as a whole.

Non-randomised comparative studies will be assessed using the ROBINS-I tool which is based on the Cochrane risk of bias tool and was developed by Sterne et al. [25]. The ROBINS-I tool assesses bias due to confounding factors, bias in the selection of the participants into the study, bias in the classification of the interventions, bias due to departures from the intended interventions, bias due to missing data, bias in the measurement of the outcomes and bias in the selection of the reported result. Each domain obtains a risk of bias judgement (no information or low, moderate, serious or critical risk of bias) followed by an overall judgement of bias based on the responses from the signalling questions within each domain [25].

Single-arm studies will not be quality assessed but will be characterised according to key features, such as size, patient group, patches used, outcomes collected and the nature of the follow-up.

### Evidence synthesis and reporting

A scoping search identified a paucity of data, with few comparative studies conducted to date exploring the types and ranges of patches available, in the specific patient population.

Statistical heterogeneity will be quantified with the *I*^2^ and *τ* statistics [24] and reasons explored as able. Small study biases will be explored using funnel plots if there are sufficient studies (10 or more) [24].

If a meta-analysis of the randomised comparative studies is possible, we will use a random effects method in Stata version 14.0 using the metan command. Sensitivity  analyses will assess  differences associated with study design and patient population where feasible. Data will be analysed on a intention to treat  basis without imputation of missing data where possible. Given the nature of the comparison, no cluster randomised nor cross-over trials are anticipated.

It is unlikely that a meta-analysis can be performed given the anticipated relatively low number of comparative studies and differences in methodology; in this circumstance, the methods and results of the systematic review will be written up in a qualitative manner. A narrative summary of the evidence will be produced and where appropriate tables will report the study design, patient population, intervention details and outcomes for each patch identified in the review. An overall comparison of the use versus non-use of patch augmentation will be carried out if possible. Evidence will be sub-grouped according to patch type.

Economic and cost evaluations will be narratively summarised.

## Discussion

There is a pressing need to improve the surgical options available for rotator cuff repairs in order to improve tendon healing and patient outcomes. Evidence on the use of patches is limited and only covers a subset of those patches available as only a small number of comparative studies have been completed. The main aim of this review is to collate the current evidence base regarding the use of patches to augment rotator cuff surgery. This in turn will help to identify those patches frequently used in practice and help assess the study designs used in previous and ongoing clinical trials. The results of this review will help to develop, using consensus methods, the design of a definitive randomised trial assessing the effectiveness and cost-effectiveness of a patch to augment surgical repair of the rotator cuff that is both acceptable to stakeholders and is feasible.

## Additional files

Additional file 1: PRISMA-P Checklist. Completed PRISMA-P checklist to confirm compliance to the reporting standards of review protocols. (DOCX 29.8 kb)

Additional file 2: Embase Search Strategy. Search strategy detailing all the MeSH headings and search terms used to identify the relevant studies in Embase using the Ovid platform. (DOCX 16 kb)

## Abbreviations

AEs: Adverse events
ASES: American Shoulder and Elbow Surgeon
CENTRAL: Cochrane Controlled Register of Trials
CRD: Centre for Reviews and Dissemination
DARE: Database of Abstracts of Reviews of Effects
DASH: Disabilities of the Arm Shoulder and Hand
EQ-5D: EuroQoL-5D
FDA: Food and Drug Administration
HRQoL: Health-related quality of life
HUI: Health Utilities Index
MHRA: Medicines and Healthcare products Regulatory Agency
NHS: National Health Service
OSS: Oxford Shoulder Score
PARCS: Patch Augmented Rotator Cuff Surgery
PRISMA: Preferred Reporting Items for Systematic Reviews and Meta-Analyses
PRO: Patient-reported outcome
RCT: Randomised controlled trial
SF-36: Short Form 36
SPADI: Shoulder Pain and Disability Index
SST: Simple Shoulder Test
UCLA: University of California at Los Angeles
VAS: Visual analog scale
WHO: World Health Organization
