# Supplementary material for: Systematic review of the surgical management of rotator cuff repair with an augmentative patch: a feasibility study protocol
Source: Syst Rev. 2018 Nov 13;7:187. doi: 10.1186/s13643-018-0851-1 (PMC6234662; doi:10.1186/s13643-018-0851-1)
Supplement: Supplementary file 2 — Embase Search Strategy. Search strategy detailing all the MeSH headings and search terms used to identify the relevant studies in Embase using the Ovid platform. (DOCX 16 kb) [file 13643_2018_851_MOESM2_ESM.docx]

**Additional file 2: Embase Search Strategy**

| [# ▲](http://ovidsp.tx.ovid.com/sp-3.24.1b/ovidweb.cgi?&S=PLMLFPJGMLDDBEIANCHKCHOBGBHDAA00&Sort+Sets=descending) | **Searches** | **Results** |
| --- | --- | --- |
| 1 | exp rotator cuff rupture/ | 5732 |
| 2 | exp rotator cuff injury/ | 8726 |
| 3 | exp tendon injury/ | 20086 |
| 4 | (rotator cuff tear or rotator cuff rupture or rotator cuff injur* or supraspinatus tear or supraspinatus rupture or supraspinatus injur* or infraspinatus tear or infraspinatus rupture or infraspinatus injur* or subscapularis tear or subscapularis rupture or subscapularis injur* or tendon tear or tendon rupture or tendon injur* or shoulder pain or shoulder injur*).mp. | 38603 |
| 5 | or/1-4 | 39799 |
| 6 | exp tissue repair/ | 17952 |
| 7 | exp shoulder surgery/ | 4863 |
| 8 | (tissue repair or shoulder surgery or augment*).mp. | 212917 |
| 9 | exp tissue scaffold/ | 11559 |
| 10 | exp extracellular matrix/ | 99144 |
| 11 | exp bioprosthesis/ | 7140 |
| 12 | exp allograft/ | 40591 |
| 13 | exp autograft/ | 13831 |
| 14 | exp surgical mesh/ | 12582 |
| 15 | exp acellular dermal matrix/ | 1282 |
| 16 | (tissue scaffold or extracellular matrix or bioprosthesis or allograft or autograft or surgical mesh or acellular dermal matrix or "GraftJacket" or "Zimmer Collagen Repair Patch" or "Permacol" or "TissueMend" or "BioBlanket" or "Conexa" or "AlloPatch" or "Shelhigh Encuff Patch" or "OrthADAPT" or "Restore" or "CuffPatch" or "Polytape" or "SportMesh" or "Arthelon" or "Gore-tex" or "BioFiber" or "STR Grafts" or "Lars Ligament" or "X-repair").mp. | 321825 |
| 17 | or/6-16 | 533208 |
| 18 | 5 and 17 | 3914 |
| 19 | Clinical trial/ | 1042043 |
| 20 | Randomized controlled trial/ | 481803 |
| 21 | Randomization/ | 84993 |
| 22 | Single blind procedure/ | 29844 |
| 23 | Double blind procedure/ | 141452 |
| 24 | Crossover procedure/ | 55396 |
| 25 | Placebo/ | 333461 |
| 26 | Randomi?ed controlled trial$.tw. | 155939 |
| 27 | Rct.tw. | 23487 |
| 28 | Random allocation.tw. | 1668 |
| 29 | Randomly allocated.tw. | 27352 |
| 30 | Allocated randomly.tw. | 2233 |
| 31 | (allocated adj2 random).tw. | 860 |
| 32 | Single blind$.tw. | 19262 |
| 33 | Double blind$.tw. | 178171 |
| 34 | ((treble or triple) adj blind$).tw. | 698 |
| 35 | Placebo$.tw. | 254195 |
| 36 | Prospective study/ | 401796 |
| 37 | Retrospective study/ | 538437 |
| 38 | Longitudinal study/ | 109396 |
| 39 | (Case control adj (study or studies)).tw. | 103770 |
| 40 | (Cohort adj (study or studies)).tw. | 187964 |
| 41 | or/19-40 | 2619904 |
| 42 | 18 and 41 | 794 |
| 43 | limit 42 to dd=20060401-20170228 | 370 |
